# Prevalence of common bacterial STI pathogens and the microscopic diagnostic approach to abnormal vaginal discharge in a tertiary care hospital in Bangkok, Thailand

**DOI:** 10.1371/journal.pone.0331668

**Published:** 2025-09-08

**Authors:** Chenchit Pichailuck, Piyachat Sakunborrirak, Rossaphorn Kittiyaowamarn, Chanon Neungton, Pareeda Taunwaena, Natnaree Girdthep, Rungnapa Luengprasit, Pochamana Phisalprapa, Chayanis Kositamongkol

**Affiliations:** 1 Unit of Sexual Medicine, Department of Obstetrics and Gynaecology, Faculty of Medicine Siriraj Hospital, Mahidol University, Bangkok, Thailand; 2 Bangrak STIs Center, Division of AIDS and STIs, Department of Disease Control, Ministry of Public Health, Bangkok, Thailand; 3 Department of Nursing, Faculty of Medicine Siriraj Hospital, Mahidol University, Bangkok, Thailand; 4 Department of Medicine, Faculty of Medicine Siriraj Hospital, Mahidol University, Thailand; The University of Sydney, AUSTRALIA

## Abstract

In Thailand, sexually transmitted infections (STIs) persist as a significant public health issue, notwithstanding the affordability of treatments. The primary challenge lies in diagnostic methodologies. According to the Thai National Treatment Guidelines for abnormal vaginal discharge, wet preparation using proportion of white blood cell (WBC) counts and epithelial cell (EC) guides presumptive STI treatment. This study investigated the prevalence of common STI pathogens in sexually active women presenting with abnormal vaginal discharge and WBC > EC under microscopy; and the cost-minimization analysis of this approach. A cross-sectional study was done during July 2021–March 2023 at the Siriraj Female STI Clinic, Bangkok, Thailand. The eligible participants were non-pregnant Thai women aged 18–50 years with the following conditions; being sexually active in prior one year, presenting with abnormal vaginal discharge, having WBC > EC under microscopy, and no allergy to cefixime and azithromycin which were the presumptive treatment in the study. The endocervical swabs were sent for molecular diagnosis of STI pathogens (polymerase chain reaction; PCR). Cost-minimization analysis comparing two approaches, PCR and wet preparation, was done. From an initial 199 participants, 186 were eligible. The average age was 31.1 ± 9.4 years and their sex debut was at 19.7 ± 3.8 years. Around 10% of them, sex partners had STIs. Prevalent STI pathogens included *C. trachomatis*(20.4%), *N. gonorrhoeae*(7.0%), *M. genitalium*(5.9%) and *T. vaginalis*(3.8%). Presumptive treatment yielded no severe immediate or delayed adverse effects. Using wet preparation as a primary test with presumptive treatment, cost per one case cured was almost three times lower than that of using PCR as a primary test (1250.0 vs 3454.4 Thai Baht). In summary, a quarter of the sexually-active Thai women with abnormal vaginal discharge and WBC > EC under microscopy had either *C. trachomatis* or *N. gonorrhoeae*. The use of wet preparation-guided presumptive STI treatment is practical and cost-saving, compared with the PCR approach. **Trial registration**: Thai Clinical Trial Registry (TCTR20210702001, 2 July 2021).

## Introduction

Abnormal vaginal discharge is a common presenting symptom in gynaecological clinics and often results from vaginal dysbiosis or sexually transmitted infections (STIs) [1–2]. While dysbiosis is more prevalent, STIs exert greater healthcare strain [1]. The World Health Organization (WHO) reported an upsurge in STIs, especially among young people. In 2020, 374 million new STI cases were estimated, with trichomoniasis (41.7%), chlamydial cervicitis (34.5%) and gonorrhoea (21.9%) predominating. This trend incurs significant economic costs due to its subclinical nature, expanding sexual networks, vertical transmission and long-term sequelae [3]. Thailand has witnessed a similar increase in STIs, particularly among 15–24-year-olds [4]. Untreated STIs can cause severe health issues such as pelvic inflammatory disease, chronic pain, infertility and ectopic pregnancy [5]. During pregnancy, they heighten the risk of preterm birth and labour. Many infected individuals are asymptomatic, complicating detection and treatment [6].

Prompt diagnosis and treatment are pivotal for STI control [2]. However, in resource-scarce areas, high-cost molecular diagnostic methods, such as nucleic acid amplification tests, are often prohibitive and lack the ability to produce immediate results. Studies indicate that Gram staining is a viable diagnostic method, linking a white blood cell (WBC) count ≥30/1000x with *C. trachomatis* infections [7–8], and with an even lower threshold of ≥5/1000x found in a Thai female sex worker cohort [9]. Nevertheless, the limited availability and lack of standardization of Gram staining hinder its broader use [10].

Wet preparation is quicker, more cost-effective, and more suited to all Thai healthcare levels than Gram staining. The method is also covered in medical education and supported by online resources [11]. Wet preparation contains a comparable diagnostic yield for common female reproductive tract infections [2] and, like Gram staining, no STI pathogen grading system [12]. However, wet preparation may appear more influenced by sample preparation technique, subjectivity and durable record. In 2022, the Royal Thai College of Obstetricians and Gynaecologists introduced guidelines for managing abnormal vaginal discharge in reproductive-aged women, recommending same-day nucleic acid amplification testing, bedside wet preparation and a syndromic approach [2]. A higher WBC-to-epithelial cell (EC) ratio, especially WBC ≥ 30/400x, indicates the presence of STI pathogens in sexually active women. The syndromic approach is limited to mobile units due to concerns about antibiotic overprescription.

In general, young people or those with multiple sex partners are considered the population at risk for STI [10]. The present study demonstrated the prevalence of common bacterial STIs in all reproductive-aged women who presented with abnormal vaginal discharge and WBC > EC under microscopy. Additionally, as Thailand has limited resource of molecular diagnostic tool, we assessed the cost-minimization of wet preparation as the primary test for STI treatment initiation, focusing on the prevalence of four common pathogens—*C. trachomatis*, *N. gonorrhoeae*, *M. genitalium* and *T. vaginalis*.

## Materials and methods

### Study design and setting

This cross-sectional study was conducted at the Siriraj Female STI Clinic from July 2021 to March 2023. The study was approved by the Siriraj Institutional Review Board (reference Si-1064/2020) and registered with the Thai Clinical Trial Registry (TCTR20210702001, 2 July 2021).

### Population

The inclusion criteria were age 18–50 years, having unprotected sexual intercourse in prior one year; no cervical lesions, and WBC > EC under microscopy. Patients with allergy to cefixime and azithromycin were excluded. Those with inconclusive or invalid polymerase chain reaction (PCR) results were withdrawn from the study.

### Study protocol and data collection

All eligible participants were explained about the study. Those who agreed to participate filled up the written consent form. They underwent sexual risk behaviour assessments, pelvic examinations with a dry speculum and specimen collection. The specimens were examined using vaginal pH measurement, wet preparation and PCR, which was the gold standard for diagnosing each STI in this study. The PCR detected seven pathogens, including *C. trachomatis*, *N. gonorrhoeae*, *M. genitalium*, *M. hominis*, *U. urealyticum*, *U. parvum* and *T. vaginalis*. However, current consensus has shown that *M. hominis*, *U. urealyticum* and *U. parvum* are not clinically relevant STIs and should not be treated [13]. However, these pathogens are included in the commercial test package being used in the study. After providing consent, the participants were placed in the lithotomy position, and a vaginal speculum was reinserted for endocervical swab collection. The swab, rotated three times in the cervical os, was stored in modified Minimum Essential Medium at 4–8°C for weekly PCR testing.

In accordance with the Centers for Disease Control and Prevention (CDC) 2021 guidelines [10], presumptive treatment for *N. gonorrhoeae* and *C. trachomatis* began immediately in women with likelihood of STI acquisition. Participants with WBC count ≥30/400x were given azithromycin (1 g) and cefixime (800 mg) and monitored for at least 15 minutes for side effects. Those with concurrent trichomoniasis, candidiasis, or bacterial vaginosis received metronidazole (2 g) or fluconazole (200 mg) at enrolment [2].

To prevent reinfection and protect the vaginal ecosystem, the patients were advised against vaginal intercourse until PCR results were available. Delayed side effects were assessed at the two-week follow-up. PCR results guided tailored treatment for non-gonococcal/non-chlamydial infections: metronidazole 2 g for 1 day for trichomoniasis; and for *M. genitalium*, a 7-day course of doxycycline 100 mg twice daily plus azithromycin 1 g for 1 day, followed by 500 mg for 3 days [2]. STI management included partner notification and treatment for *C. trachomatis*, *N. gonorrhoeae*, *T. vaginalis* and *M. genitalium* as routine practice [2].

### Outcome measurement

#### Pelvic examination and vaginal pH.

The diagnosis of pelvic inflammatory disease involved assessing cervical motion tenderness, uterine tenderness during palpation, or adnexal tenderness by pressing areas adjacent to the uterus, with any of these signs indicative of pelvic inflammatory disease. Vaginal pH was measured using a pH paper with the scale of 0.5 (MQuant, Germany). It was measured by applying pH paper to discharge on a dry speculum, with a pH ≥ 5 indicating abnormal.

#### Wet preparation.

In the wet preparation procedure, a cotton swab was used to collect vaginal discharge from the posterior and lateral fornix of participants in the lithotomy position. The swab was then immersed in 1 mL of saline solution for immediate light microscope. At 100x magnification, the presence of *T. vaginalis* was discerned by a size comparable to that of a WBC and its characteristic jerky motion. The application of 10% potassium hydroxide revealed pseudohyphae, signifying *C. albicans*, and thus indicative of vaginal candidiasis.

At 400x, the specimen was evaluated for WBC count for at least 3 random fields to retrieve the average number of WBC count as previous evidence linking higher WBC counts with chlamydial infection [7]. (S1 File. Method of wet preparation)

#### PCR analysis for seven organisms.

This study investigated seven STIs, including *C. trachomatis*, *N. gonorrhoeae*, *M. genitalium*, *T. vaginalis*, *M, hominis, U. urealyticum* and *U. parvum.* Endocervical swabs and their 2 mL of transfer media were stored at 4–8°C before PCR. The swabs were equilibrated to ambient temperature and vortexed. A 200 µL aliquot from each sample was processed for DNA extraction using the QIAamp DNA Mini Kit (Qiagen, Hilden, Germany) per the manufacturer’s protocol.

The Anyplex^TM^ II STI-7e Detection Kit (Seegene, Seoul, Korea) facilitated multiplex real-time PCR for the pathogens, which was conducted according to the manufacturer’s instructions in a CFX96 thermocycler (Bio-Rad; Hercules, CA, USA). The PCR mixture included 5 μL of DNA, 4x STI-7 TOM and Anyplex^TM^ PCR Mix, giving a total of 20 μL. Thermal cycling involved 4 minutes of UDG activation at 50°C and 15 minutes of pre-denaturation at 95°C. This was followed by 50 cycles of 95°C for 30 seconds, 60°C for 1 minute and 72°C for 30 seconds, with melting temperature analysis from 55°C to 85°C (5 seconds/0.5°C). Process controls were added pre-extraction to ensure DNA extraction and PCR efficacy.

The AnyPlex^TM^ II STI-7e had good agreement for the detection of *C. trachomatis* and *M. genitalium* with the standard-of-care diagnostic methods, Cohen’s kappa of 0.87 [95% confidence interval; CI 0.82–0.92] and 0.80 [95%CI 0.74–0.87], respectively. There was lower agreement for the detection of *N. gonorrhoeae* and *T. vaginalis*, at 0.37 [95%CI 0.19–0.55] and 0.52 [95%CI 0.25–0.80], respectively [14].

#### Presumptive treatment and side effects.

All eligible participants received a single dose of azithromycin (250 mg, 4 tablets) and cefixime (100 mg, 8 tablets). The participants were monitored for immediate side effects for at least 15 minutes at the clinic. Delayed side effects were assessed at the 2-week follow-up.

#### Cost-minimization analysis.

The cost minimization analysis was done after all PCR results were retrieved. All eligible participants had WBC count ≥10/400x; and the prevalence of STI pathogens was not different between WBC count 10–29/400x and ≥30/400x. Therefore, to address the variability in WBC thresholds across guidelines, we calculated the cost per cured patient with a conservative WBC cut-off ≥10/400x, aiming to minimize misdiagnosis risks associated with higher WBC counts. Analysing from a societal perspective, we compared two approaches: (1) ‘presumptive treatment’ (initiating therapy based on WBC > EC under microscopy) and (2) ‘PCR-first’ (delaying treatment until PCR confirmation). Drawing on the 97–98% effectiveness rates reported in other studies, we assumed a 100% efficacy rate [15–16].

The standard presumptive treatment involved the oral administration of cefixime 800 mg for *N. gonorrhoeae* and azithromycin 1 g for *C. trachomatis*. Other STI pathogens that must be treated are *M. genitalium* (doxycycline 100 mg PO BID for 7 days, followed by azithromycin 1 g PO OD for 1 day, then azithromycin 500 mg PO OD for 3 days) and *T. vaginalis* (metronidazole 2 g PO single dose) [2]. As treatment of *M. hominis*, *U. urealyticum* and *U. parvum* is not recommended, it is excluded from the analysis [13].

The analysis incorporated direct medical costs (diagnostic tests, medications, healthcare services) and direct non-medical expenses (food, travel). Costs were derived from the Standard Cost List for Health Technology Assessment [17], and adjusted to 2022 Thai Baht using the consumer price index. Median reference drug prices were obtained from Thailand’s Ministry of Public Health’s Drug and Medical Supply Information Center (Table 1) [18].

**Table 1:** Standard costs for health technology assessment in Thailand.

	Approaches		Cost (2022,THB)	References
	Presumptive treatment	PCR-first		
**First visit**				
Antibiotics^a^				Calculation
* N. gonorrhoeae*	Yes	No	89	Drug and Medical Supply Information Center, Ministry of Public Health
* C. trachomatis*	Yes	No	36	Drug and Medical Supply Information Center, Ministry of Public Health
Healthcare service				
* *OPD service	Yes	Yes	388.8	Standard cost lists for Health Technology Assessment
* *Pelvic examination	Yes	Yes	149.9	Standard cost lists for Health Technology Assessment
* *Wet smear	Yes	Yes	74.4	Standard cost lists for Health Technology Assessment
PCR	No	Yes	2000	Siriraj Hospital
Food	Yes	Yes	158.3	Standard cost lists for Health Technology Assessment
Travel	Yes	Yes	58.3	Standard cost lists for Health Technology Assessment
**Second visit**				
Antibiotics^b^				Calculation
* N. gonorrhoeae*	No	Yes, if positive	89	Drug and Medical Supply Information Center, Ministry of Public Health
* C. trachomatis*	No	Yes, if positive	36	Drug and Medical Supply Information Center, Ministry of Public Health
* T. vaginalis*	No	Yes, if positive	2.9	Drug and Medical Supply Information Center, Ministry of Public Health
* M. genitalium*	No	Yes, if positive	100	Drug and Medical Supply Information Center, Ministry of Public Health
Healthcare service				
* *OPD service	Yes	Yes	388.8	Standard cost lists for Health Technology Assessment
* *Pelvic examination	Yes	No	149.9	Standard cost lists for Health Technology Assessment
* *Wet smear	No	No	74.4	Standard cost lists for Health Technology Assessment
PCR	Yes if symptoms persit	No	2000	Siriraj Hospital
Food	Yes	Yes	158.3	Standard cost lists for Health Technology Assessment
Travel	Yes	Yes	58.3	Standard cost lists for Health Technology Assessment
**Third visit**				
Antibiotics^a^				Calculation
* N. gonorrhoeae*	No	No	89	Drug and Medical Supply Information Center, Ministry of Public Health
* C. trachomatis*	No	No	36	Drug and Medical Supply Information Center, Ministry of Public Health
* T. vaginalis*	Yes, if positive	No	2.9	Drug and Medical Supply Information Center, Ministry of Public Health
* M. genitalium*	Yes, if positive	No	100	Drug and Medical Supply Information Center, Ministry of Public Health
Healthcare service				
* *OPD service	Yes	No	388.8	Standard cost lists for Health Technology Assessment
* *Pelvic examination	No	No	149.9	Standard cost lists for Health Technology Assessment
* *Wet smear	No	No	74.4	Standard cost lists for Health Technology Assessment
PCR	No	No	2000	Siriraj Hospital
Food	Yes	No	158.3	Standard cost lists for Health Technology Assessment
Travel	Yes	No	58.3	Standard cost lists for Health Technology Assessment

Cost-minimization analysis was done based on the fact that each patient visited the Clinic no more than three times. Calculation was done using the standard costs for health technology assessment in Thailand, including healthcare service, antibiotics, food and travel.

^a^Presumptive treatment.

^b^Antibiotics for *N. gonorrhoeae*: cefixime 800 mg PO single dose; for *C. trachomatis*: azithromycin 1 g PO single dose; for *T. vaginalis*: metronidazole 2 g PO single dose; for *M. genitalium*: doxycycline 100 mg PO BID for 7 days, followed by azithromycin 1 g PO OD for 1 day, then azithromycin 500 mg PO OD for 3 days.

OPD, out-patient department; PCR, polymerase chain reaction; THB, Thai Baht

The economic outcomes are presented as total costs and costs per one case cured. Additionally, cost of over-medical prescription and direct cost saved (excluding medication) in presumptive treatment were calculated. The cost of over-medical prescription equals to total drug cost of presumptive treatment minus that of PCR-first approach, and the direct cost saved was calculated using the same manner.

#### Sample size calculation and statistical analysis.

Descriptive statistics were used to summarize the data as frequencies (n, %), 95% CI, means (SD) and medians (range). Categorical variables were compared using Fisher’s exact test or the chi-squared test, and Student’s t-test or the Wilcoxon rank-sum test was applied to parametric and non-parametric data, respectively. Analyses were performed using Stata Statistical Software, version 12 (StataCorp LLC, College Station, TX, USA).

The sample size was estimated from a previous study of American women, in which 21.8% of those with a gram-stained WBC ≥ 30/400x had chlamydial cervicitis [7]. With an alpha of 0.05 and a margin of error of 0.07, the required sample size for patients with a WBC ≥ 30/400x was 139. All consecutive participants with WBC > EC under microscopy were enrolled during the study period.

## Results

**Fig 1:**
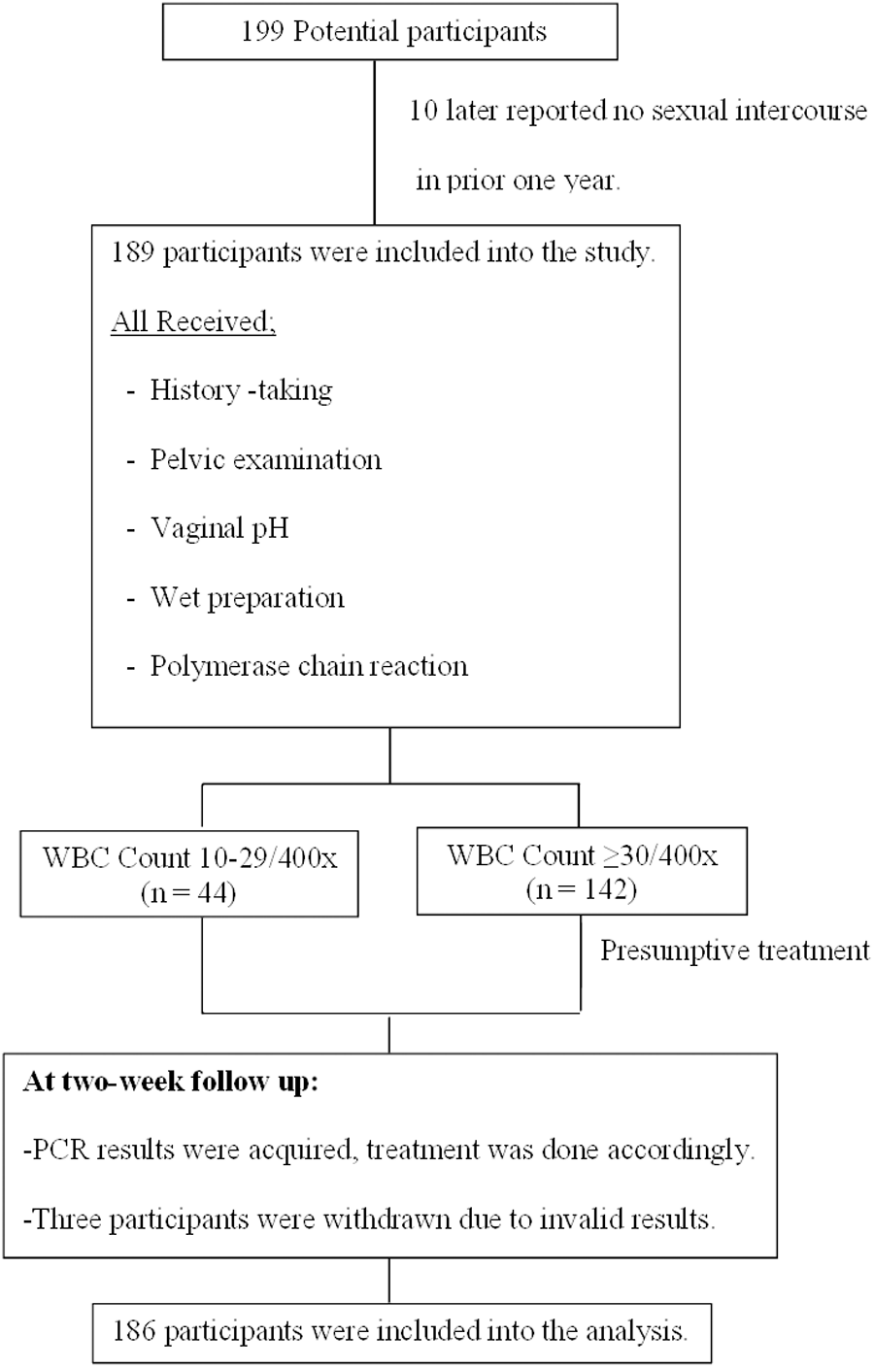
Study flow. From 199 potential participants, ten were excluded due to later report of no sexual activities in the last year and three were withdrawn due to invalid polymerase chain reaction results.

**Fig 2:**
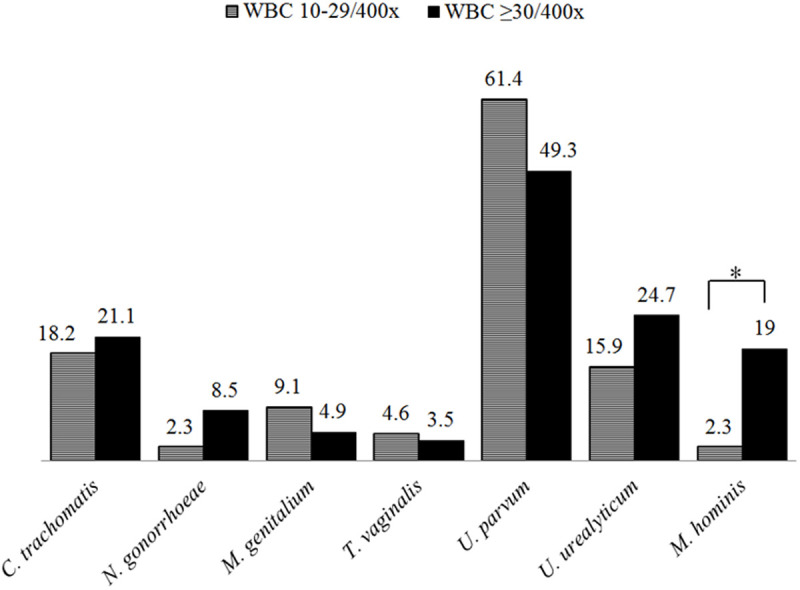
Prevalence of each detected pathogen being divided by group of WBC counts. There was no statistical difference between WBC 10-29/400x and WBC ≥ 30/400x except for *M. hominis* (P 0.007).

Among the 199 potential participants, 10 were excluded due to later report of no sexual activity in the last year, and three were withdrawn due to invalid PCR results. All eligible participants had ≥ 10 WBCs/400x, including 44 patients with WBC 10–29/400x (23.7%) and 142 with WBC ≥ 30/400x (76.3%). (Fig 1) Prevalent STI pathogens included *C. trachomatis* 38/186 (20.4; 95%CI 14.9–26.9), *N. gonorrhoeae* 13/186 (7.0; 95%CI 3.8–11.7), *M. genitalium* 11/186 (5.9; 95%CI 3.0–10.3) and *T. vaginalis* 7/186 (3.8; 95%CI 1.5–7.6). Prevalence of each detected organism divided by groups of WBC count was demonstrated in Fig 2. Pathogen prevalence did not significantly differ between groups of WBC counts.

The characteristics of the participants were shown in Table 2. The mean age was 31.1 ± 9.4 years, the body mass index was 22.1 ± 4.0 kg/m^2^, and the age at sexual debut was 19.7 ± 3.8 years. Over half had a bachelor’s degree. Approximately 20% had a history of miscarriage, and a quarter had a history of STIs. Of them, 10% had multiple partners in the last 6–3 months. Of their partners, 17 reported a history of STIs. The characteristics of participants with or without *C. trachomatis* were comparable whereas those with *N. gonorrhoeae* were older, started sexual activities at later age and contained a higher prevalence of trichomoniasis. Co-occurrence of *C. trachomatis* and *N. gonorrhoeae* with vaginal candidiasis was high, at 15.8% and 23.1%, respectively. (Table 2)

**Table 2:** Characteristics of participants and STI pathogens which are covered by the presumptive treatment (N = 186).

		PCR for *C. trachomatis*			PCR for *N. gonorrhoeae*		
	Total (n = 186)	Negative (n = 148)	Positive (n = 38)	P	Negative (n = 173)	Positive (n = 13)	P
**Age** (y)	31.1 ± 9.4	31.0 ± 9.4	31.3 ± 9.3	0.887	30.8 ± 9.4	34.9 ± 8.3	0.126
<25	58 (31.2)	46 (31.1)	12 (31.5)	0.993	56 (32.4)	2 (15.4)	0.264
25-35	75 (40.3)	60 (40.5)	15 (39.5)		70 (40.4)	5 (38.5)	
>35	53 (28.5)	42 (28.4)	11 (29.0)		47 (27.2)	6 (46.1)	
**Body mass index** (kg/m^2^)	22.1 ± 4.0	21.9 ± 4.0	22.9 ± 4.0	0.175	22.3 ± 4.1	20.0 ± 2.5	0.047
**Education**				0.828			0.084
None/elementary	16 (8.6)	12 (8.1)	4 (10.5)		15 (8.7)	1 (7.7)	
High school/vocational	65 (35.0)	51 (34.5)	14 (36.9)		64 (37.0)	1 (7.7)	
Bachelor’s or higher	105 (56.4)	85 (57.4)	20 (52.6)		94 (54.3)	11 (84.6)	
**Sex debut** (y)	19.7 ± 3.8	19.5 ± 3.6	20.2 ± 4.5	0.327	19.5 ± 3.7	22.2 ± 4.3	0.012
**Partners**							
Partners in 3 mo ≥ 2	19 (10.2)	14 (9.5)	5 (13.2)	0.502	16 (9.3)	3 (23.1)	0.112
Partners in 6 mo ≥ 2	26 (14.0)	20 (13.5)	6 (15.8)	0.718	23 (13.3)	3 (23.1)	0.327
Partners had STIs	17 (9.1)	15 (10.1)	2 (5.3)	0.353	16 (9.3)	1 (7.7)	0.851
**History of miscarriage**	32 (17.2)	24 (16.2)	8 (21.1)	0.481	28 (16.2)	4 (30.8)	0.179
**Having child/children**	71 (38.2)	55 (37.2)	16 (42.1)	0.576	67 (38.7)	4 (30.8)	0.569
**History of any STIs**							
Herpes genitalis	13 (7.0)	10 (6.8)	3 (7.9)	0.806	13 (7.5)	0	0.305
Trichomoniasis	5 (2.7)	3 (2.0)	2 (5.3)	0.271	5 (2.9)	0	0.537
Pelvic inflammatory disease	9 (4.8)	7 (4.7)	2 (5.3)	0.891	8 (4.6)	1 (7.7)	0.619
Anogenital warts	29 (15.6)	23 (15.5)	6 (15.8)	0.970	27 (15.6)	2 (15.4)	0.983
Syphilis	6 (3.2)	2 (1.4)	4 (10.5)	0.004	6 (3.5)	0	0.495
**Concurrent symptoms**							
Pruritus	120 (64.5)	100 (67.6)	20 (52.6)	0.086	111 (64.2)	9 (69.2)	0.713
Genital lesions	35 (18.8)	29 (19.6)	6 (15.8)	0.592	33 (19.1)	2 (15.4)	0.743
Pelvic pain/fever	34 (18.3)	29 (19.6)	5 (13.2)	0.360	31 (17.9)	3 (23.1)	0.643
**Pelvic examination**							
Cervical motion tenderness	5 (2.7)	4 (2.7)	1 (2.6)	0.981	5 (2.9)	0	0.534
Uterine tenderness	5 (2.7)	4 (2.7)	1 (2.6)	0.981	2 (2.9)	0	0.534
Adnexal tenderness	6 (3.2)	6 (4.1)	0	0.207	6 (3.5)	0	0.495
**Vaginal pH ≥ 5**	110 (59.1)	87 (58.8)	23 (60.5)	0.845	101 (58.4)	9 (69.2)	0.443
**Wet preparation**							
Pseudohyphae favoring vaginal candidiasis	52 (28.0)	46 (31.1)	6 (15.8)	0.061	49 (28.3)	3 (23.1)	0.684
*T. vaginalis*	2 (1.1)	1 (0.7)	1 (2.6)	0.297	1 (0.6)	1 (7.7)	0.016

The characteristics of participants with or without *C. trachomatis* were comparable whereas those with *N. gonorrhoeae* were older, started sexual activities at later age and contained a higher prevalence of trichomoniasis.

BV, bacterial vaginosis; PCR, polymerase chain reaction; STIs, sexually transmitted infections

Table 3 compares the costs per cured patient between the presumptive treatment and PCR-first methods. With all treatments assumed to be 100% effective, the cost per cured patient was 2.76 times lower for the microscope-approach with presumptive treatment. The cost of over-medical prescription in presumptive treatment was 20 825.20 Thai Baht. However, the direct cost that could be saved by implementing presumptive treatment was 430 835.31 Thai Baht, compared to the PCR-first approach. Table 4 shows the detailed cost incurred by presumptive treatment and PCR approach.

**Table 3:** Cost-per-case-cured comparison between two approaches.

Approaches	# of first-line prescription	# of second treatment	# of PCR	Total cost (2022 THB)	Cost per case cured (THB)
(1) Presumptive treatment	186	16	16	232 506.8	1250.0
(2) PCR –first	60	0	186	642 516.9	3454.4

With all treatments assumed to be 100% effective, the cost per cured patient was 2.76 times lower for the microscope-approach with presumptive treatment.

PCR, polymerase chain reaction

**Table 4:** Detailed cost incurred by presumptive treatment and PCR approach. Only direct medical and direct non-medical costs were included.

PCR results	n	Costs for presumptive treatment (2022 THB)			Costs for PCR-first approach (2022 THB)		
		Drugs	Healthcare Service	Food and transportation	Drugs	Healthcare Service	Food and transportation
Negative	50	6256	30 656	10 833	–	150 094	21 666
Up	47	5881	28 817	10 183	–	141 089	20 366
Uu	15	1877	9197	3250	–	45 028	6500
Ct, Mh, Up	14	1752	8584	3033	499	42 026	6066
Ct, Up	13	1627	7971	2817	464	39 025	5633
Uu, Up	7	876	4292	1517	–	21 013	3033
Mh, Uu	5	626	3066	1083	–	15 009	2167
Ng	5	626	3066	1083	447	15 009	2167
Ct, Mh, Uu, Up	4	500	2453	867	143	12 008	1733
Ct, Ng, Up	3	375	1839	650	375	9006	1300
Mg, Uu	3	676	10 622	1950	300	9006	1300
Ng, Uu	3	375	1839	650	268	9006	1300
Ct, Mg, Up	2	450	7081	1300	200	6004	867
Mg, Tv	2	456	7081	1300	206	6004	867
Mg, Up	2	450	7081	1300	200	6004	867
Mh	2	250	1226	433	–	6004	867
Ct, Tv, Up	1	128	3541	650	39	3002	433
Ct, Uu, Up	1	125	613	217	36	3002	433
Mg, Uu, Up	1	225	3541	650	100	3002	433
Ng, Mg, Uu	1	225	3541	650	190	3002	433
Ng, Mh, Up	1	125	613	217	89	3002	433
Tv	1	128	3541	650	3	3002	433
Tv, Mh	1	128	3541	650	3	3002	433
Tv, Mh, Uu	1	128	3541	650	3	3002	433
Tv, Uu, Up	1	128	3541	650	3	3002	433
**Sum**	**186**	**24 394**	**160 881**	**47 232**	**3568**	**558 351**	**80 598**
**Total cost**		**232 506.8**			**642 516.9**		
**Total cost per patients**		**1250.0**			**3454.4**		

With all treatments assumed to be 100% effective, the cost per cured case was 2.76 times greater for the PCR-first approach. The cost of over-medical prescription in presumptive treatment was 20 825.20 Thai Baht. However, the direct cost that could be saved by implementing presumptive treatment was 430 835.31 Thai Baht, compared to the PCR-first approach.

Ct, *C. trachomatis*; Ng, *N. gonorrhoeae*; Mg, *M. genitalium*; Mh, *M. hominis*; Uu, *U. urealyticum*; Up, *U. parvum*; Tv, *T. vaginalis*

After the presumptive treatment, two participants experienced immediate nausea without vomiting. Delayed side effects included nausea without vomiting in eleven patients, diarrhoea in three patients, and one headache, with no patient seeking medical advice. The clinical cure and microscopic findings are shown in Table 5.

**Table 5:** Clinical cure and microscopic findings at 2 weeks. Of all participants, 120 were present at the two-week follow-up.

	Ct (n = 22)	Ng (n = 11)	Mg (n = 5)	Tv (n = 2)	Mh (n = 17)	Uu (n = 29)	Up (n = 58)
**Clinical cure**	13 (59.1)	8 (72.7)	4 (80)	1 (50)	10 (58.8)	15 51.7)	31 (53.5)
**Wet preparation**							
WBC < EC	10 (45.5)	6 (54.6)	2 (40)	0	8 (47.1)	12 (41.4)	25 (43.1)
WBC < EC and pseudohyphae^*^	5 (22.7)	2 (18.2)	0	0	3 (17.7)	6 (20.7)	13 (22.4)
WBC > EC	6 (27.3)	3 (27.3)	3 (60)	2 (100)	6 (35.3)	10 (34.5)	17 (29.3)
BV^**^	1 (4.6)	0	0	0	0	1 (3.5)	3 (5.2)

Clinical cure, or absence of all symptoms, was found in 59.1% and 72.7% of participants with *C. trachomatis* and *N. gonorrhoeae*, respectively. However, a quarter of each infection had persistent WBC > EC or no microscopic cure which might represent co-infection with other STI organisms.

Ct, *C. trachomatis*; Ng, *N. gonorrhoeae*; Mg, *M. genitalium;* Mh, *M. hominis*; Uu, *U. urealyticum*; Up, *U. parvum*; Tv, *T. vaginalis*; BV, bacterial vaginosis; EC, epithelial cells; WBC, white blood cells.

* Vaginal candidiasis was defined as detection of pseudohyphae under microscopy.

** Bacterial vaginosis was diagnosed using Amsel criteria.

## Discussion

Wet preparation, a simple method requiring only a microscope, glass slide and normal saline solution; can be advocated as an initial diagnostic tool for women with abnormal vaginal discharge in resource-limited settings. Despite the fact that wet preparation appears more influenced by sample preparation technique, subjectivity and durable record, it appears much more practical than other bedside diagnostic tools like Gram staining. The WBC > EC under microscopy resembles WBC count ≥10/400x. Of them, the specimens reveal that 20% had *C. trachomatis* and 7% had *N. gonorrhoeae*. Consistent with prior research in Norwegian women [19], this study supports that WBC > EC under microscopy bolsters the possibility of starting presumptive treatment. Importantly, the present study demonstrates its cost-minimization far surpasses that of the PCR-first method, highlighting its value as a primary test in settings with limited resources. Additionally, the clinical cure following this approach was up to 72.2%. This study advocates the use of the Thai National Guidelines [2] to endorse microscope as a presumptive treatment-guided tool when same-day PCR result is not applicable.

Point-of-care STI test (POCT) is the ideal strategy to mitigate STI problems worldwide [20]. Wet preparation is a promising POCT as it fulfills the definition of appropriate POCT by the World Health Organization that includes REASSURED (Real time connectivity; Ease of sample collection; Affordability; Sensitive; Specific; User-friendly; Rapid, Equipment-free; Deliverable) [21]. Rapid real-time PCR or antigen-based tests have been continuously developed [22,23]; however, the diagnostic accuracy compared with the conventional PCR in real-life practice remains far from the expectation [24]. Albeit lack of standardization for diagnosing bacterial STI-related vaginitis [12], WBC > EC under microscopy suggests vaginal and pelvic inflammatory disease-related inflammation. Moreover, wet preparation detects bacterial vaginosis and vaginal candidiasis which are the two most common causes of abnormal vaginal discharge in reproductive-aged women [1].

The current investigation supports previous studies showing that mixed vaginal infections are very common [25]. Vaginal candidiasis, often encountered in gynaecological clinics [1], is diagnosed by clinical symptoms and detected pseudohyphae under microscopy, leading to frequent antifungal use. According to the Thai Guidelines [2], the microscopic picture of acute vaginal candidiasis is EC > WBC whereas WBC > EC ratios indicate bacterial, protozoan or chronic fungal infections [2]. In our study, among those treated for candidiasis, 15.4% (8/52) had *C. trachomatis*, and 3.8% (2/52) had *N. gonorrhoeae*. Without molecular tests, such STIs are at risk of being transmitted. Implementing wet preparation as a routine bedside test could promptly address them. On top of that, a two-week posttreatment microscopic review may aid in managing the co-infections [26].

The presumptive treatment aims to cover the common infections and to stop further onward transmission. The present study shows that a quarter of sexually-active Thai women with abnormal vaginal discharge and WBC > EC under microscopy acquiring either *C. trachomatis* or *N. gonorrhoeae.* Therefore, when the diagnosis using PCR is not available, in practice, oral medication suits better as the presumptive treatment. Cefixime, an oral third-generation cephalosporin, has been used as an alternative treatment of *N. gonorrhoeae* with high efficacy at 98% [27], alongside with azithromycin for *C. trachomatis* at 97% [16]. However, the present study shows much lower clinical cure, at 72.2%. The effectiveness of this presumptive treatment regimen as well as the long-term consequences such as drug resistance should be further studied.

The prevailing presumptive treatment mainly addresses *C. trachomatis* and *N. gonorrhoeae*, yet STI panels also cover *Mycoplasma* spp. and *Ureaplasma* spp. *M. genitalium*, now acknowledged as an STI pathogen, is symptomatic in only 10–30% of cases [28]. Although *M. hominis, U. urealyticum* and *U. parvum* are often seen as commensals [29,30] and require no treatment [13], they have been associated with bacterial vaginosis [31], which is a dysbiosis connecting to infections in the upper genital tract [32] and to vaginal candidiasis [25]. Moreover, the present study demonstrates a statistically significant association between *M. hominis* and elevated WBC count. This raises a caution about its inflammation potential. Further research is needed to clarify the association between these infections and vaginal dysbiosis, as well as the consequences of such inflammatory process.

The debate around initiating presumptive treatment has been significant among healthcare providers. This study supports the benefits of presumptive treatment, showing that its costs are significantly lower (by 2.76 times) than those of precise diagnostic tests. Our direct costs, based on the Standard Cost List for Health Technology Assessment [17], indicate public sector averages, with private settings often being higher. In line with the recommendations by the Centers for Disease Control and Prevention [10], the study also noted minimal adverse events from presumptive treatment. In contrast, untreated or delayed treatment in STI patients increases transmission risks due to extensive, unregulated sexual networks. While overuse of antibiotics resulting from a syndromic approach may risk drug resistance and not reduce long-term STI prevalence [33], wet preparation offers a mitigation strategy. Nonetheless, the higher standard of STI care remains our goal by means of enhancing STI surveillance; expanding high-risk group screenings; improving clinical management for symptomatic cases; and promoting affordable, molecular point-of-care tests in Thailand.

A key strength of the study is the introduction of evidence supporting the Thai guideline to use the microscope-guided presumptive treatment. This insight is invaluable for shaping policies in resource-limited nations. The study also highlights the notable rate of co-infections at both initial and follow-up visits, underscoring the need for heightened vigilance in managing vaginitis. The single-centre nature of the study and a small representative group are our limitations, given that STI prevalence can differ regionally. However, the diversity of participants from suburban areas partly compensates for this difference. Another limitation is that there is no control arm to demonstrate the prevalence of STI pathogens in WBC < 10/400x. The baseline prevalence of the two STI pathogens in low-risk population may be required to complement the national policy development.

## Conclusion

Among sexually-active Thai women presenting with abnormal vaginal discharge, wet preparation can be an acceptable method to guide presumptive treatment, with almost three- time lower expense than using PCR-based approach. Nonetheless, this approach has pragmatic rather than diagnostic role and may be taken into consideration in resource-constraint settings. Its long-term effects, particularly drug resistance, and real-life effectiveness need to be further studied.

## Supporting information

S1 File QR code for techniques of wet preparation. (TIF)

S2 File Dataset excel file. (XLSX)

## References

[1] Chayachinda C, Thamkhantho M, Chalermchockcharoenkit A, Neungton C, Thipmontree W. Characteristics of clients at the Siriraj Female STD Clinic during 2011-2015. Siriraj Med Bull. 2018;11(3):182–9.

[2] Chayachinda C, Chinhiran K, Kittiyaowamarn R, Chaithongwongwatthana S, Teeratakulpisarn N. The Thai 2022 Sexually Transmitted Infections Treatment Guideline: Abnormal Vaginal Discharge. Thai J Obstet Gynaecol. 2022;30(4):222–33. doi: 10.14456/tjog.2022.20

[3] World Health Organization. Sexually transmitted infections (STIs). https://www.who.int/news-room/fact-sheets/detail/sexually-transmitted-infections-(stis). 2023.

[4] HIV INFO HUB. Rate of five main STIs reported case in Thailand, 2009–2022. 2023. Available from: https://hivhub.ddc.moph.go.th/epidemic.php

[5] Chayachinda C, Rekhawasin T. Reproductive outcomes of patients being hospitalised with pelvic inflammatory disease. J Obstet Gynaecol. 2017;37(2):228–32. doi: 10.1080/01443615.2016.1234439 27750467

[6] Tachawatcharapunya S, Chayachinda C, Parkpinyo C. The prevalence of bacterial vaginosis in asymptomatic pregnant women during early third trimester and the pregnancy complications. Thai J Obstet Gynaecol. 2017;25(2):96–103. doi: 10.14456/tjog.2017.15

[7] Marrazzo JM, Handsfield HH, Whittington WLH. Predicting chlamydial and gonococcal cervical infection: implications for management of cervicitis. Obstet Gynecol. 2002;100(3):579–84. doi: 10.1016/s0029-7844(02)02140-3 12220782

[8] Lusk MJ, Garden FL, Rawlinson WD, Naing ZW, Cumming RG, Konecny P. Cervicitis aetiology and case definition: a study in Australian women attending sexually transmitted infection clinics. Sex Transm Infect. 2016;92(3):175–81. doi: 10.1136/sextrans-2015-052332 26586777

[9] Arunothong S, Yasamut W, Chetacha N, Somrit J, Putthawong P. A retrospective study of relationship between white blood cell counts in cervical discharge and *Chlamydial cervicitis* among young adults: is the simple microscopy an alternative method for diagnosing the disease?. Biomedical Sciences and Clinical Medicine. 2013;52(1–2):1–10.

[10] Workowski KA, Bachmann LH, Chan PA, Johnston CM, Muzny CA, Park I, et al. Sexually Transmitted Infections Treatment Guidelines, 2021. MMWR Recomm Rep. 2021;70(4):1–187. doi: 10.15585/mmwr.rr7004a1 34292926 PMC8344968

[11] Tuangrattanasirikun D, Chayachinda C, Rachapromma P, Sonwicha S. Effect of on-line vs on-site class on medical students’ competency in vaginal wet mount. Siriraj Med Bull. 2023;16(4):271–7.

[12] Vieira-Baptista P, Almeida G, Bogliatto F, Bohl TG, Burger M, Cohen-Sacher B, et al. International Society for the Study of Vulvovaginal Disease Recommendations Regarding Female Cosmetic Genital Surgery. J Low Genit Tract Dis. 2018;22(4):415–34. doi: 10.1097/LGT.0000000000000412 29994815

[13] Horner P, Donders G, Cusini M, Gomberg M, Jensen JS, Unemo M. Should we be testing for urogenital *Mycoplasma hominis, Ureaplasma parvum* and *Ureaplasma urealyticum* in men and women? - a position statement from the European STI Guidelines Editorial Board. J Eur Acad Dermatol Venereol. 2018;32(11):1845–51. doi: 10.1111/jdv.15146 29924422

[14] Bodiybadu K, Danielewski J, Plummer E, Bradshaw CS, Machalek DA, Garland SM, et al. Comparison of Seegene AnyPlexTM II STI-7e with standard-of-care diagnostic methods for the detection of *Mycoplasma genitalium, Chlamydia trachomatis, Neisseria gonorrhoeae*, and *Trichomonas vaginalis*. Lett Appl Microbiol. 2023;76(1):ovac002. doi: 10.1093/lambio/ovac002 36688743

[15] de Vries HJC, de Laat M, Jongen VW, Heijman T, Wind CM, Boyd A, et al. Efficacy of ertapenem, gentamicin, fosfomycin, and ceftriaxone for the treatment of anogenital gonorrhoea (NABOGO): a randomised, non-inferiority trial. Lancet Infect Dis. 2022;22(5):706–17. doi: 10.1016/S1473-3099(21)00625-3 35065063

[16] Geisler WM, Uniyal A, Lee JY, Lensing SY, Johnson S, Perry RCW, et al. Azithromycin versus Doxycycline for Urogenital Chlamydia trachomatis Infection. N Engl J Med. 2015;373(26):2512–21. doi: 10.1056/NEJMoa1502599 26699167 PMC4708266

[17] Riewpaiboon A. Standard cost lists for Health Technology Assessment. http://costingmenu.hitap.net/24964710

[18] Drug and Medical Supply Information Center M of PH. Drug and Medical Supply Information Center. https://dmsic.moph.go.th/index/drugsearch/1

[19] Randjelovic I, Moghaddam A, Freiesleben de Blasio B, Moi H. The Role of Polymorphonuclear Leukocyte Counts from Urethra, Cervix, and Vaginal Wet Mount in Diagnosis of Nongonococcal Lower Genital Tract Infection. Infect Dis Obstet Gynecol. 2018;2018:8236575. doi: 10.1155/2018/8236575 30147292 PMC6083538

[20] Toskin I, Govender V, Blondeel K, Murtagh M, Unemo M, Zemouri C, et al. Call to action for health systems integration of point-of-care testing to mitigate the transmission and burden of sexually transmitted infections. Sex Transm Infect. 2020;96(5):342–7. doi: 10.1136/sextrans-2019-054358 32241905 PMC7402556

[21] Land KJ, Boeras DI, Chen X-S, Ramsay AR, Peeling RW. REASSURED diagnostics to inform disease control strategies, strengthen health systems and improve patient outcomes. Nat Microbiol. 2019;4(1):46–54. doi: 10.1038/s41564-018-0295-3 30546093 PMC7097043

[22] Gaydos CA. Review of use of a new rapid real-time PCR, the Cepheid GeneXpert® (Xpert) CT/NG assay, for Chlamydia trachomatis and Neisseria gonorrhoeae: results for patients while in a clinical setting. Expert Rev Mol Diagn. 2014;14(2):135–7. doi: 10.1586/14737159.2014.871495 24450867 PMC4061495

[23] Morris SR, Bristow CC, Wierzbicki MR, Sarno M, Asbel L, French A, et al. Performance of a single-use, rapid, point-of-care PCR device for the detection of *Neisseria gonorrhoeae, Chlamydia trachomatis,* and *Trichomonas vaginalis*: a cross-sectional study. Lancet Infect Dis. 2021;21(5):668–76. doi: 10.1016/S1473-3099(20)30734-9 33242473 PMC9884536

[24] Ha MT, Pham TL, Nguyen TA, Le VT. Performance of antigen-based rapid test for *Chlamydia trachomatis* in comparison with polymerase chain reaction test. Medicina Clínica Práctica. 2024;7(3):100447. doi: 10.1016/j.mcpsp.2024.100447

[25] Qi W, Li H, Wang C, Li H, Zhang B, Dong M, et al. Recent Advances in Presentation, Diagnosis and Treatment for Mixed Vaginitis. Front Cell Infect Microbiol. 2021;11:759795. doi: 10.3389/fcimb.2021.759795 34796129 PMC8592905

[26] Chayachinda C, Chinhiran K, Aneklap P, Rachapromma P, Sonwicha S, Neungton C. Bacterial vaginosis: A comprehensive approach to management in reproductive-aged Thai women. Thai J Obstet Gynaecol. 2023;31:318–25. doi: 10.14456/tjog.2023.36

[27] Yang KJ, Kojima N, Bristow CC, Klausner JD. Effectiveness of Cefixime for the Treatment of Neisseria gonorrhoeae Infection at 3 Anatomic Sites: A Systematic Review and Meta-Analysis. Sex Transm Dis. 2023;50(3):131–7. doi: 10.1097/OLQ.0000000000001742 36729626 PMC9906985

[28] Raj JS, Rawre J, Dhawan N, Khanna N, Dhawan B. *Mycoplasma genitalium*: A new superbug. Indian J Sex Transm Dis AIDS. 2022;43(1):1–12. doi: 10.4103/ijstd.ijstd_103_20 35846530 PMC9282694

[29] Zhang N, Wang R, Li X, Liu X, Tang Z, Liu Y. Are *Ureaplasma* spp. a cause of nongonococcal urethritis? A systematic review and meta-analysis. PLoS One. 2014;9(12):e113771. doi: 10.1371/journal.pone.0113771 25463970 PMC4252037

[30] Kang W-T, Xu H, Liao Y, Guo Q, Huang Q, Xu Y, et al. Qualitative and Quantitative Detection of Multiple Sexually Transmitted Infection Pathogens Reveals Distinct Associations with Cervicitis and Vaginitis. Microbiol Spectr. 2022;10(6):e0196622. doi: 10.1128/spectrum.01966-22 36314938 PMC9769840

[31] Bansal S, Bhargava A, Verma P, Khunger N, Panchal P, Joshi N. Etiology of cervicitis: Are there new agents in play? Indian J Sex Transm Dis AIDS. 2022;43(2):174–8. doi: 10.4103/ijstd.ijstd_75_21 36743104 PMC9890980

[32] Ravel J, Moreno I, Simón C. Bacterial vaginosis and its association with infertility, endometritis, and pelvic inflammatory disease. Am J Obstet Gynecol. 2021;224(3):251–7. doi: 10.1016/j.ajog.2020.10.019 33091407

[33] Vanbaelen T, Manoharan-Basil SS, Kenyon C. Effect of mass treatment on the long-term prevalence of gonorrhoea, chlamydia and syphilis-a systematic review. Int J STD AIDS. 2024;35(7):550–64. doi: 10.1177/09564624241239994 38506648

