# Supplementary figures and images for: Prevalence of common bacterial STI pathogens and the microscopic diagnostic approach to abnormal vaginal discharge in a tertiary care hospital in Bangkok, Thailand
Source: PLoS One. 2025 Sep 8;20(9):e0331668. doi: 10.1371/journal.pone.0331668 (PMC12416634; doi:10.1371/journal.pone.0331668)

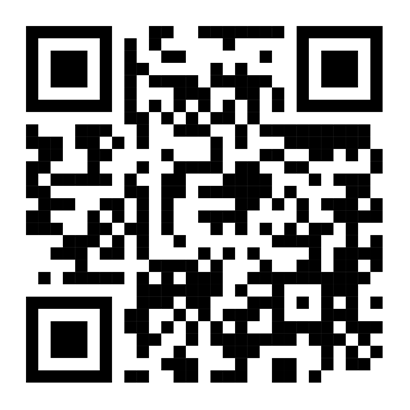

Supplement: S1 File — (TIF) [file pone.0331668.s001.tif]
